# Novum Organum Medicorum; or, New Medical Logic

**Published:** 1826-07

**Authors:** 


### VIII.

*Novum Organum Medicorum; A New Medical Logic, or the Art of Thinking and Right Reasoning applied to Practical Medicine; exhibiting the Principles advanced in a larger Work under the same Title.* By VINZENZO LANZA, M.D. Professor of Clinical Medicine in the Ospidale della Pace, at Naples. Translated from the Italian; By C. STORMONT, M.D. London; Samuel Highley, 174, Fleet Street, and Webb Street, St. Thomas's Hospital, 1826. Pp. 284.

[First Article.]

THE volume before us, is a translation of the principal part of a work, the object of which is to shew, that the science of medicine may be successfully cultivated upon the principles of induction recommended by Lord Bacon, and to exemplify the application of that method of investigation, by a minute and logical analysis of all the facts which can fall under the cognizance of a physician, in the examination of all the physiological and pathological actions and alterations, to which the human body is liable, whether they be constitutional or local, and whether they be produced by diseases or remedies. The translation is divided into two books, in the first of which are discussed, "the logical rules which it is necessary to observe, in order that the nature of diseases, and the choice of remedies may be clearly discovered, without danger of mistake." In this book, which is divided into fifteen short chapters, the author treats of experience and induction as affording, when duly combined, the only means whereby the physician can accomplish the object of his art, the cure of diseases. He gives a brief explanation of what he modestly calls, his "hypothesis," respecting the nature of diseases and the operation of remedies.

"Experience," says the author, "affords to the physician five grounds only for his reasoning, and these are the following: first, the exact investigation of the external causes which have produced the disease; secondly, the complete description of the phenomena of the disease itself, not less than of the idiosincracy of the patient; thirdly, the impartial observation of the things usually recognized as agreeable or hurtful to the patient, especially those of the non-naturals; fourthly, the result, happy or otherwise, of the cure; fifthly, the analogy, rightly instituted, between the various species of the disease itself, and between diseases similar or opposed to each other." 2.

The induction, again, which the physician employs in reasoning upon the facts derived from these sources, the author divides into two kinds, the one he calls clinical or practical, and the other theoretical or hypothetical. The former is nothing more than common reason applied to the facts for the purpose of ascertaining the true relations existing between them, and the consequences deducible from their union. Hypothetical induction, on the other hand, is described as a species of reasoning founded upon preconceived ideas of the essence of life, health and disease, and applied to any particular malady, in order to explain its nature and the operation of remedies. All reasoning upon such grounds must of necessity be hypothetical, since we can have no ideas of that nature, except what are derived from hypothesis.

The former species of reasoning is always the safest guide in practice, whether to enable us to form a correct prognosis, or to treat a disease with success and confidence. In proportion as the obscurity of the facts in any case of disease compels us to rely upon hypothetical, rather than upon clinical induction, must be the uncertainty of the prognosis and of the result of the treatment employed.

In introducing to his readers his own ideas of the "Theory of Diseases," our author remarks that those physicians who despise or neglect this distinction, and confidently rely upon hypothetical reasoning as all-sufficient in directing the practice of physic, or who employ it even in preference to practical induction, are justly censured as physicians of system, of the chair, of the pen, and as bad practitioners. To avoid this imputation, the author proposes to make use of hypothesis only where it is rendered indispensable by the absence of clear facts for the foundation of clinical induction, conceiving it to be his duty simply to explain his own theory without being overanxious whether others should think differently upon the subject.

The *vital force*, is a term whereby the author expresses merely the abstract idea of that incomprehensible thing which inhabits the living machine, and there, by its physical operations, makes it such as it is while life endures. When this force operates in the most perfect manner health is enjoyed, which is that kind of life in which living beings delight, because they can discharge all the functions essential to the full enjoyment of life. When, on the contrary, the vital force operates unduly, or in a mode different from that of health, disease is the result, which is a state of life irksome to the living being, because it suffers from the non-performance or undue performance, of its usual and necessary functions. By this distinction, according to our author, all diseases are divided hypothetically, into two great classes, viz. those of grade, which consist of a simple change of the degree or impetus of the vital force; and those of mode, which depend upon a change of the mode, as well as of the degree of the vital force.

In diseases of the former class, that is, of grade, life continues obedient to the same laws, and in the same manner as in health, differing only in the degree or impetus of the vital force. The vital force may suffer three variations of degree. It may suffer greater or less excitement than ordinary. Persons when intoxicated, over-heated, or excited by exercise, afford examples of the former, and such diseases are termed *hyperstenic*. Exhaustion from hunger, cold, or fatigue, again, afford examples of diseases from defect of excitement, which our author terms *hypostenic*. The third variety of diseases of grade depend less upon excess or defect of excitement, than upon disturbances of the vital force. A thorn wounding a nervous filament, caustic acting upon the skin, a tooth in the act of cleaving the gum, worms in the bowels, indigestible substances in the stomach, the fœtus wedged in the passage, the placenta retained in the uterus, and humours stagnating or extravasated, produce an irregular commotion in the excitement of the vital force, which is called irritation, and diseases of this kind are denominated *irritative*.

Diseases of grade are not numerous, and they are so slight as scarcely to be called diseases, being simple disturbances of health, which are easily restored when the obstacles are removed.

In diseases of mode, on the contrary, life acquires new qualities. Such diseases arise, run their courses and terminate according to laws in all respects peculiar to their natures: and, whether excitant or debilitant remedies be employed, they are not deprived of their morbid qualities, except by the return of health, or in the event of death. Thus angina, pleurisy, rickets, scrofula, the gout, small pox, schirrus, are not simple deviations from, or disturbances of the healthy vital actions, but possess, each of them, essential and peculiar morbid qualities, since, although some of them may be closely akin to each other, yet there is always in each a distinguishing quality, that prevents their being confounded.

Diseases of mode do not admit of being classed with respect to degree, because diseases of grade, even when they change into diseases of mode, become immediately hyperstenic; as, for example, should torpor from cold terminate in pneumonia, the hypostenic nature of the disease is instantly changed into hyperstenic. Modal diseases are divisible into *diathestic*, or those which leave the body more disposed to them, such as angina tonsillaris; and *adiathestic*, or those which destroy entirely, or greatly diminish the susceptibility or disposition to them, such as small pox or measles.

Many diseases are complicated so as to exhibit the phenomena of hypostenia, hyperstenia, irritation, diathestic modality, and adiathestic modality more or less united in the same individual case, except only that hypostenia and hyperstenia never can be co-existent at the same time in one person. Thus to a patient affected with an endemic intermittent, may be superadded a petechial fever with worms, and he may, at the same time, be extremely weak and emaciated, from the long duration of his complaint. Such a person presents a physiological debility, or hypostenia in the state of his body, symptoms of irritation peculiar to vermination, the phenomena distinctive of the morbid petechial adiathestic modality, and those of the diathestic modality peculiar to the periodical endemic.

Our author divides remedies into four classes, according to their mode of operation, naming them *specifics*, *vivificants*, *solvents*, and *irritants*. With respect to *specifics* he says:

"We admit that in every agent there is a specific quality, or a particular mode of operation, whence the remedies (even the most analogous between themselves, as opium and wine) differ not less in degree than in mode, so that by increasing or diminishing, in whatever way, the dose of the one, we never come to produce precisely the effect which is proper to the other: but whether it be that they are rare or that we are not acquainted with them, very few indeed are the remedies which can be used as specifics, that is for that particular medicinal quality in them which directly corrects the morbid quality, and, on this account, the greatest part of remedies come to be used for their power of increasing or diminishing the impetus of the vital force, whence, if they do not remove the disease, yet they diminish its impetus." 10.

*Vivificants* are those agents which yield life its pabulum, elevate its phenomena, and quicken its force. *Solvents* are those which impoverish life, diminish the vividness of its phenomena and enfeeble its force. This meaning, attached to the Italian word *scioglenti*, is so very different from what usage has conferred upon the kindred English word *solvents*, that the translator appears to have been a good deal embarrassed in finding an English term to convey exactly the ideas comprehended by the Italian word *scioglenti*. He informs us, in the preface, that the author includes blisters, electricity, magnetism, cicuta, hyosciamus, digitalis, laurocerasus, belladonna, and even alum and iron, in the class of *scioglenti*, while he says that opium is classed with wine, among the *vivificanti*. It would hardly have been consistent with propriety, for the translator to use the English term *reductives*, as a name to embrace all the abovementioned medicines. Conceiving the meaning attached to the word scioglenti, to be conformable to that of the Latin verb, *solveo*, to solve, loosen or untie the hold which a disease has of the body, or in short to promote the resolution of a disease, the translator has generally employed the words *relaxant* or *relaxants*, instead of *solvent* or *solvents*. We think that *resolvents* would have more nearly conveyed the author's meaning.

With respect to *irritants*, our author observes, that the vital force has a *capacity* for receiving the influence of external agents; that there is a certain point, to the extent of which, every agent operates regularly, as a vivificant, or as a solvent, but that, if given in doses exceeding the natural capacity, it disturbs, irritates and disorders life: so as, instead of proving of benefit, to produce a new disease of irritation, which, whatever may have been the nature of the agent, whether solvent or vivificant, may assume the character of inflammation, congestion, nervous affection, or any other form of disease whatever.

The vital capacity to receive any remedy, or the measure beyond which any agent may prove irritant, depends; 1. Upon the constitution, idiosyncrasy, age, sex, and habits of the patient: for instance, upon an abstemious person, wine acts as an irritant, not as a vivificant, in like manner as vinegar, nitre or antimony act as irritants upon other persons. 2. Upon the specific nature of the disease. Thus an agent, which usually operates "*ordinately*," may be an irritant in certain diseases, or in certain stages of diseases, as water in hydrophobia, and as the bark occasionally does in ague. 3. Upon the nature of the remedies themselves, it being constantly observed that the most efficacious are the most apt to prove irritant.

Hence the necessity of rules of practice, not only to form correct indications, but to prescribe remedies discreetly and moderately, in order to ensure their "ordinate" operation, whether they be vivificants or solvents, in all except the rare case, in which it is desirable to use irritants: for whatever may be the nature of a remedy, and how much soever it may be indicated, it must, if given in excess, produce the effect of an irritant, exasperate the disease, or occasion new diseases in the same manner as any other remedy, which is counter-indicated, would do.

In conclusion, the author states, that it is not his intention to support his theory in the present work by any attempt at demonstration, except so far as its fitness to assist in forming practical inferences may be shewn incidentally in those parts of the work, where he may find the use of it necessary. That it is consistent with the soundest principles of philosophy he means to prove in a future work on the elements of theoretic medicine. The principal distinctive feature of this theory, as our readers will perceive, consists in the admission of the modality or particular qualities of diseases and of remedial agents. Brown was the author of the hypothetical principles according to which it is imagined, that the vital force can suffer no change, except in degree, and our author points out in the course of this work, numerous instances of the great practical errors into which this erroneous doctrine has led its votaries.

He proceeds in the third and subsequent chapters of the first book to treat of clinical induction, as applied to the investigation of the external causes of diseases; to the phenomena of diseases, and the constitution of the patient; to the discovery of the juvantia and lædentia; to the result of the treatment; to medical analogy; to the distinction between morbid states and morbid processes: of hypothetical induction as applied to account for the hyperstenical nature of all morbid processes; of the cure of diseases in general; of clinical induction applied particularly to the specific cure of diseases; to the minorative or gradal cure of diseases; to the palliative cure; and to the prophylactic cure of diseases; and, lastly, of the prognosis in general.

The Second Book, which is divided into fourteen chapters, and occupies 223 of the 284 pages composing the volume, is devoted to the consideration of "General Morbid Forms." The author's observations on these are arranged into chapters, on inflammation, obstruction and congestion; on fever; on resolution and other changes which take place in fever, inflammation, obstruction and congestion; on the cure of fever, inflammation, obstruction, and congestion in general; on nervous complaints; on a bad habit of body; on morbid alterations of the temperature, the excretions and colour of the body; on wounds and the healing process; on commotion or concussion, distension and pressure; on suppuration; on ulcers; on organic blemishes; on gangrene; and on death.

With respect to the causes of diseases, the first of the five sources of facts from which, according to our author, the nature of diseases may be deduced, the Brownonian doctrine regards them as the principal foundation of the physician's judgment. According to this theory—the causes of all diseases must be excitant or debilitant; and, consequently, every disease must be either hyperstenic or hypostenic; and, since pain suffered during disease tends to debility, even hyperstenic diseases must, in process of time, spontaneously become hypostenic. This principle seems, at first sight, so simple and clear as to obtain the fullest confidence from the inexperienced, but has not only not been followed but loudly condemned by practical physicians as leading continually to the most dangerous mistakes. The principal reasons which shew that the contemplation of the external causes is not sufficient to determine the nature of diseases are 1, That the knowledge of the causes depends, for the most part, not on the observation of the physician, but on the information derived from the patient, which is often inexactly given, either because all the causes may not be known to him, or that he may have motives for wishing to conceal some of them. 2, That the external causes of the greater number of diseases are not simple, but of opposite kinds, and may have operated together or alternately, so that it must depend, for the most part, on the will, or on the preconceived hypothesis of the physician, more than on any fixed rule, which shall be adopted in preference. 3, That in the majority of diseases medical science has not yet determined their causes, or their mode of operation.

Our author distinguishes diseases as regards their etiology into those dependent upon the continued operation of their causes, and those that are independent of the causes that excited them. For instance, torpor from cold may be removed by the properly graduated application of heat, or the removal of the cause. Exhaustion from hunger may be remedied by the due administration of food. The irritation occasioned by worms may be removed by their expulsion from the bowels, or that from teething by freely dividing the gums. In such cases the diseases are dependent on the continued operation of the causes which gave rise to them, and are hyperstenic, hypostenic, or irritative, according to the nature of those causes. But in pneumonia or hepatitis, whether they may have been excited by heat or cold, anger or fear, it is to be observed, that the disease is invariably the same, and, at the same time, that it proceeds in its course independently of the removal of the causes which produced it.

If the knowledge of the nature of the causes of diseases be necessary to determine the nature of those diseases that are dependent thereon, it is equally useful to account for the violence, obstinacy, and danger of those which are independent of their causes, and to this valuable purpose, as our author remarks, "our good ancestors in the science made the etiology principally subservient in such diseases." The greater or less violence of the causes, the greater or less noxiousness of their nature, the longer or shorter time they may have operated, the slow and imperceptible, or rapid and striking manner in which they may have acted, are circumstances affording us essential assistance in the formation of just conclusions as to the probable severity, duration, and results of diseases.

In treating of clinical induction as applied to the phenomena of diseases, our author, after adverting in terms of reprobation to the mischief that has resulted from what he calls the greatest of Brown's errors, namely, his confounding the physiological state of the patient with the pathological nature of the disease, or inferring from the patient's debility that the disease must be hypostenic, distinguishes diseases, as regards their phenomena, into two classes, namely, those of simple and those of double phenomenology.

The former consist simply of an excess, defect or disturbed state of the healthy forces. They are exactly those diseases which were previously described as dependent upon their causes. They exhibit none but physiological phenomena: whereas, a disease of double phenomenology uniformly presents phenomena proper to itself, whatever may be the physiological state of the forces, or of the patient's constitution, for, although the activity of the disease may vary, in some degree, with the variations of the patient's strength, yet it never loses its own essential characteristics, nor does it depend on the debility of the patient, for, even when convalescence has commenced, and the disease has disappeared, the physiological debility is often at the height, and in other cases, such as phthisis, when the physiological forces of the patient are almost exhausted, the activity of the pathological actions continues unimpaired. Diseases of double phenomenology are the same as those that were described as independent of their causes. They have one set of symptoms, belonging to the physiological state of the patient, which are variable, and another set, consisting of the phenomena proper to the disease, which are constant, whether the patient be strong or weak.

From these observations, the justice of which cannot be disputed, our author takes occasion to remark, that his hypothesis regarding the nature of diseases derives confirmation, since diseases, which are independent of their causes, and in which the pathological phenomena are always the same, whatever may be the state of the patient's strength, must be new conditions effected by the morbid action of the vital force, and dependent upon a new quality or modality of that force.

In the fifth chapter, on the application of clinical induction to the discovery of the juvantia and lædentia, Professor Lanza admits the great advantage of the information to be derived from this source, for enabling a physician to ascertain the nature of diseases. In estimating the good or harm done by the means used, it is necessary to be acquainted with the regular and natural course of the disease.

In this view, he divides diseases into two classes. Those which are dependent on the continued operation of their causes, and exhibit simply physiological phenomena, namely diseases of grade, have no necessary or essential course; but are merely passive, diminishing or increasing in degree with the physiological forces of the patient: so, that in order to judge of the effects of any thing that is used in these diseases, it is only necessary to have regard to the effects produced on the physiological state of the patient. As these diseases are in their nature dependent on their causes, so their course is entirely regulated by the means employed, whence it is logically inferred, that they have no necessary or imprescriptible course. Diseases of mode, which are independent of their causes, and of double phenomenology, have, on the contrary, a necessary course: so that, increasing or diminishing the physiological strength can only increase or diminish their violence; but, whatever means may be used, they continue their course, increasing or diminishing as their pathological nature requires. If gastritis be excited, whether in consequence of fasting, of being over-heated, or of the irritation occasioned by worms, it must have a necessary increase or decrease, the excess of which may, perhaps, be mitigated by remedial agents, but the course of the disease cannot be prevented by force,

And, as such diseases are independent of the physiological state of the patient, it often happens, that, although there be great physiological debility present, yet that means increasing the strength prove hurtful, and those diminishing it still further prove beneficial.

In order to judge correctly of the relations existing between these diseases and the effects of the means employed, our author adopts the rule given by Hippocrates, but which was rejected by Brown and his followers; viz. to pay strict attention to the *physiological tolerance* which the body may appear to have for the things used, and the *alleviation of symptoms* which they produce. *The physiological tolerance of debilitants* is the ease with which reductive means, or solvents are borne, without the patient being so much debilitated by them, as might have been expected if they had been used in health, accompanied with an intolerance of excitants, so that these do not increase the physiological strength, as they would have done in a state of health. The *physiological tolerance of excitants* is, of course, the reverse. *The pathological alleviation of symptoms* is the diminution of the weight of the morbid phenomena proper to the disease, either absolutely considered, or relatively to the extent these might have increased, had the means not been employed. This diminution of these phenomena is advantageous, although it may not have been obtained without a greater reduction of the physiological strength. To render this rule the more clear our author takes typhus as an example in illustration, being a disease which the Brownonians regarded as one of great debility and hypostenic. Supposing the disease to have existed a number of days, and that there is great physiological debility, he says the following observations may be made, with a view to arriving at correct conclusions relative to the effects of the juvantia and lædentia. 1, The patient lies in bed, immersed in the vapour of his body, secluded from free access of fresh air, fasting, or subsisting on light and fluid food. This regimen is borne easily, and the reverse would be hurtful. 2, This debilitant regimen, bloodletting, and resolvent remedies are required. The disease itself produces certainly a degree of physiological debility, but less weakness is produced by the means employed than would occur in an individual in health, if he were exposed to the same treatment. 3, If the patient should clothe himself, expose himself freely to the contact of the air, move about, and eat and drink, he would not derive from such means so great an accession of physiological strength as we might be led to expect. 4, By the debilitating means used the physiological debility is increased; but the heat, febrile aridity, and redness are diminished, as well as the density of the urine, delirium, coma, and convulsions; or, at least, if these symptoms are not diminished they increase more slowly. 5, All the excitants in the world cannot prevent the disease from producing the physiological debility; they can only cause it to be somewhat less; but, while they tend to prevent the increase of debility, they increase the heat, the febrile aridity, the load on the tongue, the density of the urine, the delirium, coma, convulsions, &c. From these facts the author infers, in obedience to what he calls practical reason or common sense, and in defiance of the hypothetical reasoning of the Brownonians, that typhus is an hyperstenic disease, and that it requires to be treated by the solvent (reductive) method. He contends that, by this rule, common sense is superior to every hypothesis for determining whether diseases require exciting or debilitating treatment, and states, that, to be enabled to judge with the greatest nicety of what is useful or hurtful, we ought to attend less to the use of medicines or other things of obscure or doubtful agency, than to the effects produced by those things which are ordinarily employed for the support of life, in order to observe whether they prove useful or hurtful, or whether they increase or reduce the strength.

From the consideration of this part of his subject the author remarks, that the facts afford farther support to his hypothesis of the modality of the vital force in diseases of mode, which have a necessary course prescribed to them by their nature, and which are susceptible of alleviation, even by means which increase the debility of the natural force. He regards such diseases, as new conditions, or, as the effects of new actions of the vital force dependent on a modification assumed by that force, and curable by such means as never fail to produce disorder, when employed in the ordinary state of life, that is, in health.

Our author considers the right application of practical induction to the result of the treatment of diseases as attended with much difficulty. The self-love of the physician is apt to feel hurt, when the slender weight of his operations is fairly exhibited in the balance: while his practice is apt to be rendered pusillanimous by the recollection of unfavourable results, or audacious by events appearing in a light too favourable to his efforts. With respect to their terminations, diseases of grade, which are those dependent on their causes, of single phenomenology and of a non-necessary course, are also necessarily dependent on the means employed, for if, in those of excess in the degree of the vital force, the means employed regularly diminish it, while in those of defect they regularly increase it, and in those of irritation they restore its equilibrium, such diseases must necessarily terminate fortunately; and if, on the contrary, the operation of the exciting causes is continually increased, such diseases must become worse and worse till death ensues. Diseases again, which, according to our author's hypothesis, are termed modal, and which are independent of their causes, of double phenomenology, and have a necessary course, have a termination not necessarily dependent on the treatment employed, for, even when treated in the most proper manner they sometimes terminate in death, and though treated erroneously, yet a certain proportion will recover. For example, of a hundred patients affected with typhus who shall be treated upon the excitant method, twenty shall die, but still eighty recover; and, of the same number treated by a solvent (reductive) method, ninety shall recover, but still ten shall die. It might happen, too, that if two cases were selected for an example, the debilitant method used in the one might not prevent death, and the excitant method in the other might end in recovery. "By this," says our author "the quacks profit, by this the vulgar are often deceived." He comes to this conclusion, that that method of treatment, which has been easily borne by the patient, and which has produced alleviation of the symptoms must have been beneficial, although the disease may have gone on to an unfortunate termination; and, on the other hand, that that treatment, which has been distressing and oppressive to the patient, must have been bad, although health may have supervened under it.

With respect to the fifth source of facts for the reasonings of physicians, medical analogy, our author observes that it is liable to lead to very fallacious results if not carefully interpreted. In diseases of grade he draws from analogy the same conclusions that the Brownonians do, namely, that in the hypostenic the phenomena are all hypostenic, while in the hyperstenic they are all hyperstenic, and that in irritative diseases they are neither hyperstenic nor hypostenic, but variable as are the causes of irritation.

In diseases of mode, however, our author protests against the abuse to which Brown applied analogy. From regarding all diseases as of grade, he fell into the error of drawing conclusions as to their nature from the character of their physiological phenomena, while he overlooked their special pathological symptoms, conclusions too, which led directly to the most absurd and dangerous practice. For example, in a case of pleurisy there are, in the first place, phenomena indicative of increased vigour; in the course of the disease, the excess is observed declining into defect of vigour; and, in its advanced stages, and even from the first in some cases, there may be very great physiological debility. From observing the phenomena of vigour or debility in this disease to be analogous to the ordinary hyperstenic and hypostenic phenomena, Brown infers that pleurisy may be hyperstenic or hypostenic, and that, though hyperstenic at its commencement it may, in its course, become hypostenic.

From this conclusion our author appeals to common sense, which, at the bed-side, is called clinical induction. Observe the pathological phenomena of pleurisy, the pain, the difficult lying down, the oppression, the cough, the spitting, the dryness, the heat, the fever, and, however they may have varied with the varying strength of the patient, they altogether present a most visible identity in all the species, and in every stage of the disease. From these facts practical induction infers, that pleurisy is, from its pathological nature, an identical and incommutable disease, whatever may be the condition of the physiological strength of the patient, and however that strength may vary in the course of the disease. Hence, induction comes to a precept which, under different terms, physicians of all ages have acknowledged, that in diseases of mode, since the phenomenology is double, there must be a double analogy, one to determine, as in diseases of grade, the physiological strength of the patient, and another for comparing the pathological part of a disease, with that of other diseases, in order to determine their points of resemblance and difference, and to draw safe conclusions, as to the analogies they bear to one another.

In concluding this part of his subject our author exclaims, "can it be possible not to see the phenomena to be duplicate in these last (modal) diseases? Can it be possible not to perceive the pathological part, always identical, to be different from the physiological part, always variable? And what mind shall not judge the pathological part, in these diseases, to be a change of mode, or a new quality of life?"

Our author distinguishes diseases into "morbid states" and "morbid processes." The former are exactly what have been already described as diseases of grade, which differ from health only in the vital force being increased, diminished or disturbed, and which have no necessary course, but cease, as soon as their causes cease to operate, or the proper remedies are applied. A morbid process is a change of the processes of life, which possesses a peculiar and invariable character, independently of the causes that may have produced it, which, besides the physiological phenomena, presents pathological phenomena peculiar to itself and unknown in health, and which has a necessary course, and continues in defiance of whatever means may be employed by man to put a stop to its progress. A morbid process "consists in a series of newly ordained events, concealing, within itself, the cause of its nature, origin, and course." This part of the author's views is illustrated by the following example. Let three fingers of the same hand be treated thus; place the index in ice, the middle finger in boiling water, and the ring finger on nettles, and withdraw them in a few moments. In the first, the healthy excitement will be diminished; in the second, increased; and in the third, disturbed or irritated. If, on withdrawing the fingers and applying proper treatment, these morbid affections begin to decrease, and in a few minutes cease altogether, the changes that had existed are "morbid states;" but if, from the bad disposition of the fingers, from the strong or long continued action of the causes, or any other circumstance, there should arise a true inflammation, this is an "inflammatory process," concealing within itself the cause of its identical character, and of the arrangement of its phenomena. The scalding of the middle finger by boiling water, and the irritation of the ring finger by nettles have also the appearance of inflammation, but they are simply "inflammatory states," because they preserve the characters of diseases of grade. For this reason, when any disease is observed to have its phenomena arranged in process, it is always a sure indication that it has become a disease of mode.

Morbid processes are of two kinds. The first is called *resolvable*, or *process of simple alteration*, and occurs when life, while it changes the qualities, preserves the elements of the old or sane textures, as in inflammation, obstruction, &c. If a fit remedy be employed, or a spontaneous cure takes place, this kind of process may be resolved, and the parts remain as in health. The second kind of morbid process is termed *unresolvable*, or *process of degeneration*, and occurs when life, under its new modification, produces parasite growths of new textures, such as scirrhus, sarcoma, &c. which do not admit of resolution.

The author considers morbid processes as affording a strong argument in support of his theory of the modality of the vital force in diseases, because, as he says, those diseases which visibly alter the organic textures, must depend upon an alteration in the mode of the vital processes of life.

Our author conceives that the principal superiority of what he calls the modern Italian doctrines, over the hypothetical practice of Brown, consists in having discovered and demonstrated that morbid processes are all invariably hyperstenic; that is, that all diseases of mode, whatever differences may exist between them and a state of health, nevertheless agree amongst themselves, in having the excitement elevated in degree above the standard of health. This condition he terms the hyperstenic identity of diseases of progression. The practical proof of the hyperstenic nature of each disease of process is referred to the place where he is to treat of such diseases individually, but assuming it here as a fact the author takes occasion to apply hypothetical induction to explain it. This explanation is as follows: the body, which, according to his hypothesis, is liable to changes in the mode as well as in the degree of life, when it has suffered any change of mode, or when a morbid process is established, necessarily must sustain a new life, a new mode of being, manifested by peculiar phenomena, and even by a new texture. These morbid vital processes are consequently always supplemental, (or other lives superadded) to the ordinary life. This is made evident, by considering the physiological debility of the patient, for the art of medicine, when not possessed of specific remedies for any disease, is obliged to undertake what our author terms the simple minorative cure, that is, to reduce the degree of any morbid process, and, for this purpose, has properly recourse to reductive remedies, which, by withdrawing the pabulum of life, are also hostile to the growth of every morbid vital process.

To such as may have a difficulty in comprehending what he means by the modality or change in the quality of life, our author says, that when speaking of diseases of mode, he does not pretend to understand, or explain how the vital force changes its qualities, but merely to express the abstract idea of the phenomena presented to his observation, in the same manner as, when a weight falls from above, and is seen to accelerate its motion in proportion to the degree of its descent, we cannot understand what increases the force that urges it downwards, though we can express the abstract idea of the phenomenon, by saying that the gravity of the falling body increases every instant in degree. In like manner, he says, that diseases of a nature independent of that of their causes, of double phemonology, of a necessary course, of a termination not necessarily determined, of an invariable pathological analogy, with a growth of new texture; and a power to change the dispositions of the machine to their own nature, differ from health, not in degree alone, but also in possessing a specific nature and qualities distinct from each other as well as from health; and saying that morbid processes are independent, immutable, specific, and always hyperstenic, is the same as to say, in abstract terms, that the vital force must have assumed a new mode or quality.

Our author divides the cure of diseases in general into three kinds, the radical, palliative and prophylactic. The radical cure he divides into two kinds, the specific or modal, and the minorative or gradal. By the specific cure, we cut short the progress of a disease, by means of some agent which changes the morbid mode to health. When, as is most frequent, we possess no specific agent for the cure of a disease, we employ the minorative or gradal method of cure, consisting of vivificants when the morbid excitement is below the healthy standard, or reductive remedies, when the morbid excitement is in excess. By this method, the degree, or impetus of a disease is "minorated" or reduced as much as possible, so that the morbid quality, being deprived of its pabulum, gradually and spontaneously disappears, and health returns.

The palliative and prophylactic cures are each of two kinds, namely, analogous, or contrary to the radical cure. When analogous to the radical, they tend to alleviate or prevent a disease, while at the same time, they are useful for diminishing its impetus or degree. When contrary to the radical cure, they tend to increase the degree or impetus of a disease, even while they suspend or mitigate some urgent symptom.

In simple diseases of grade, the specific cure consists in removing the cause, the continued operation of which keeps up the disease. It was well observed by Brown, that a man exhausted by fasting, might be restored by music, or the sight of a fine woman, so far as to exert and raise himself; and our author remarks, that the minorative cure of the exhaustion produced by hunger may consist in music, love, or any other exciting passion, which may diminish the effect of hunger and prolong life, but, that food alone can produce a specific cure, so that, if this be withheld, death cannot be averted. The necessity of specific agents for the cure even of gradal diseases, shows evidently, that though they differ from health only in degree, they are really different in mode from one another.

In irritative diseases, certain agents may somewhat soothe the irritation, diminish the uneasiness, and ward off some of the worst effects of the disease, but they cannot produce a specific cure. In diseases of mode a specific cure is produced by those agents which have the property of neutralizing or dispelling the morbid cause present in adiathestic diseases, or of changing the condition of the vital force in diathestic diseases.

By a specific remedy, our author means a remedy, which, when administered at a proper time and opportunity, cures the disease completely, and without fail: which, without any delay, stops the course of a disease, so that, if given during the increase, the disease ceases to advance to its height, as it otherwise would have done, but begins to decrease as soon as the remedy begins to take effect: which is followed by the disappearance of the disease in conformity to the time, the dose, and the manner in which the medicine has been given: and which has the effect of leaving no symptom or effect of the disease undiminished.

In illustration of the minorative or gradal cure of diseases our author says, that we would bleed a man labouring under intoxication if we apprehended apoplexy; that, if it were possible to extract the wine or to neutralize it, we should effect a specific cure of drunkenness, but that bleeding is only the minorative cure of the effects. In this mode of cure, it is necessary to beware of producing too rapid and sudden a change from one state of excitement to the opposite. Heat suddenly applied to a limb torpid from cold, is apt to induce gangrenous inflammation.

In irritative diseases, it is not possible, either by raising or lowering the excitement, to restore the disturbed vital force to order, while the irritating cause continues to act. Art can only alleviate the pain, and prevent some consequences of such diseases, namely, morbid processes, such as inflammation, nervous affections, &c. which, when once established, do not cease, although the irritating cause be removed. For this reason, irritative diseases do not admit of a minorative cure. They can be relieved by the specific cure, viz. the removal of the causes, or the palliative, whereby the symptoms are alleviated, or the prophylactic, whereby morbid processes may be prevented from supervening, or may be rendered less severe and less protracted, so that time may be afforded for the cause to pass away naturally, or to be removed by art.

In diseases of mode, or morbid processes, the minorative cure consists in withdrawing the ordinary stimuli, and in applying reductive remedies, in order, that, by reducing the physiological force, the morbid vital process may be arrested in its course or destroyed. This is the method practised in the majority of diseases, for, by reducing their impetus, we afford an opportunity for the morbid vital process, after being exhausted according to those laws, to which every vital process is liable, to terminate naturally. This illustrates that valuable maxim of the ancients, which taught them to regard medicine as the follower, or handmaid, not the comptroller of Nature. The author here again laments the capital practical error of the Brownonians, who, disregarding the modality of morbid vital processes, and maintaining all diseases to be simple changes in the grade of the vital force, imagined, that by merely raising or lowering the excitement, the specific cure of all diseases might be accomplished without the aid of any particular specific remedies: and he lays down the following maxim as the proper rule for the management of the minorative cure of diseases of process.

"The lowering of the forces in morbid processes, ought never to be short of what is required by the violence and nature of the disease, nor greater than what can be easily and conveniently borne by the patient."

With respect to the palliative cure, our author remarks that, when it is analogous to the radical, there is no room for comment, since its propriety is admitted by all schools and all physicians. For example; no physician would hesitate about a local bleeding, or an emollient cataplasm, to sooth local pain, or any other symptom of a morbid process, for the cure of which general reductive measures may be required. The propriety of adopting a palliative cure, when it is contrary to the radical, is less apparent, but still may be vindicated. Thus opium alleviates pain in pleurisy and other hyperstenic diseases, but the evil that results from its use in pleurisy, shews that it has acted as an excitant, that the disease is hyperstenic, and that the mitigation of the pain has been procured by a remedy contrary to that required for eradicating the disease. By tonic astringents, we may suppress a flux, which is a symptom of an hyperstenic disease, but the consequence is, that the disease is subsequently still more exasperated.

This fact is explained according to our author's theory, as follows: the morbid pleuritic excitement differs in degree, as well as in kind, from that of the opium which, however, is also morbid and hyperstenic, and which produces the stupefaction or lulling of the pain. When the two operate at once, the excitement of the opium is overcome, and though the pleuritic hyperstenia may be increased, yet the pain is lulled by the narcotic.

This kind of cure, however, is rarely admissible, since a transient alleviation might bring with it irreparable mischief; yet it sometimes happens, that the palliative effect obtained, is greater than any possible future harm, and in such a case, it is the duty of a discreet physician, who knows exactly how to calculate the effect of the means he employs, to take advantage of it. "The great Sydenham" is justly mentioned by our author as conspicuously successful in the discriminating use of this method of cure.

The following case, which the author gives in illustration of this mode of using palliatives, being almost the only case described in the whole volume may be interesting.

"We employed opium in this manner, with the consent of our respected colleagues, in a case of lumbago, which afflicted a respectable personage. The pain was so acute and obstinate, that the patient could at no time remain in bed with sufficient tranquillity to admit of relaxant (resolvent) diaphoretics, nor could he leave his bed to take a bath, or to obey the action of purgatives. The hyosciamus proved ineffectual, and local bleeding insufficient. The fifth day passed without fever, and without any symptom menacing phlogosis of any internal organ. With the obstinacy of the pain, the other hyperstenic symptoms did not fail to increase. Opium was given liberally, so as to lull the pain. On the sixth day, the bath, diaphoretics and purgatives, could be administered without inconvenience; sufficient evacuations were obtained. On the seventh there was no longer lumbago."

In this manner opium is often used for mitigating pain in syphilis, cancer, colic, wounds, &c.

The prophylactic cure, like the palliative, may be analogous, or contrary to the radical. Remedies which are employed to prevent the paroxysm, or the return of any disease, may be analogous, or contrary to those which are fit to be employed on the attack, or during a paroxysm: for example, blood-letting may be useful to cure a palpitation of the heart, and at the same time to prevent paroxysms of that affection; but the bark and stimulants, which are so useful in preventing the paroxysms of periodical fevers, would be most injurious if given during the paroxysm. In the cure of such fevers our author maintains, that the bark operates in a manner contrary to that of the remedies which are proper for the radical cure of the paroxysms. The prophylactic method of cure must be employed with great caution in the treatment of diseases which do not admit with impunity of their paroxysms being suppressed or prevented, as is sometimes the case with gout.

Our author founds general rules for forming a prognosis in diseases on the consideration of the constitution of the patient, the occasional causes, the proximate cause, the seat, the form, transmutations, and characters of diseases, and from those things, which, being exterior with respect to the body and the disease, have still an essential influence on the results.

A disease is always to be considered more dangerous in children, aged persons, and in bad habits of body, whether from being weakened by other diseases, or the long continuance of that, regarding which, our prognosis is under consideration.

A disease is dangerous in proportion to the power or violence of its occasional causes, and to the length of time they may have been applied.

The prognosis depends chiefly on the correctness of our knowledge of the proximate cause, and of the obstacles to be expected in eradicating it. The diagnosis determines whether a disease is susceptible of a radical, minorative, palliative or prophylactic cure, and the prognosis announces what shall be the event of the disease, if left to its natural course, and what benefit may be expected from art.

The result or effects of a disease varies according as the organ in which it is seated is more or less important, and according to the form which the disease assumes. An inflammation, an obstruction, a congestion, a nervous affection, and a profuse discharge, are different forms by which organs may be affected, and, by the assistance of our knowledge of anatomy and physiology, we judge of the form of the affection, and of the probable result.

Most diseases are capable of changing their seats and forms, and such transmutations may be beneficial or hurtful. A knowledge of their varieties is necessary to the establishment of a safe prognosis.

A disease may be of so malignant a character as to defy the power of medicine, and in another case, the same disease may be so mild as to require little or no aid from medicine. The prognosis in particular cases, must be just in proportion to the powers of discrimination, and the experience of the practitioner.

A patient may derive, not only great comfort, but relief essential to his recovery from the skill of his physician, the care of his nurse, and other circumstances foreign to the nature of his disease, but in the formation of a prognosis, all such circumstances, as they may influence the result of a case, must be taken into consideration by a prudent practitioner.

We have thus endeavoured to lay before our readers a general view of the principles which Professor Lanza proposes for guiding the reasonings of physicians respecting diseases and the operation of remedies, and which he considers as calculated to lead to safe and just conclusions if followed up in practice with fidelity, care, and judgment. There can be no doubt of the advantage of proceeding to the investigation of diseases upon a systematic method, and that it is of the utmost importance to every practitioner to have his mind tutored in early life to the observance of certain rules, as to the order in which he proceeds in his enquiries into the circumstances of whatever cases are confided to his superintendence and management. We have no objection to the Novum Organum Medicorum, or System of Medical Logic of Professor Lanza, and we are apt to think that much less pains is bestowed upon this department of medical education in this country than its importance deserves. It is of course undeniable, that logic or the art of right reasoning is a universal one, and that any argument that is correct in itself, must be so, whether the subject relates to medicine, or to any other department of human knowledge; nor, after all the study that can be conferred upon logic, can it be said to be any thing more than the application of sound common sense to discover the true relations which the objects of our enquiries bear to each other: but the obscurity of the facts which form the ground on which medical men are compelled to build their conclusions, is often such as to require the nicest habits of discrimination to avoid errors, and the difficulty of the subject is often so great, that, after the greatest pains are bestowed upon it, we are still far from obtaining proofs on which the mind can rest with satisfaction. This very difficulty is apt to engender habits of carelessness or indifference in practitioners, whose zeal to reach perfection is small, or in whose characters indolence is a predominant failing. It cannot be doubted, however, that, although we cannot supply the want of common sense, by any artificial system of logic, the careful study of that science, with a view to its methodical application to the subjects of medical knowledge, must tend powerfully to guard against the bad consequences of erroneous conclusions, while, by impressing the mind of the student with the value of thinking rightly, and by exercising that faculty, he may be trained to most useful habits, that shall prove highly important to his success as a practitioner, and to his reputation, in case he should have occasion to appear before the public in the character either of an author, or of a witness. Great original talents cannot be the worse for cultivation, and a complete knowledge of the art of logic, the weapon which we employ in estimating the nature and results of diseases, may contribute as much to the soundness of our conclusions, as a knowledge of the natural and chemical qualities of medicines does to our success in prescribing them.

For these reasons, we regard the study of logic in its application to medical science, as displayed in the volume under review, as highly deserving the attention of all who wish to form for themselves habits of reasoning with correctness, and of avoiding errors in the practice of their professional duties. We shall defer making any observations on the merits or defects of Professor Lanza's theoretical views, as developed in this publication, until after we shall have examined the application of his logical method to the consideration of General Morbid Forms, as exemplified in the second book, or division of the volume, to which we shall proceed in the next number of this Journal.

[To be continued.]

